# Zeolitic Imidazolate Framework-8 (ZIF-8) as a Carrier for Kaempferol Delivery to Protect Against Gamma Radiation-Induced Mortality and Damage

**DOI:** 10.3390/pharmaceutics17111489

**Published:** 2025-11-18

**Authors:** Gang Yang, Jing Wang, Rong Wang, Lu Han, Chunai Gong, Jiyuan Chen, Minyan Chen, Yongfang Yuan

**Affiliations:** Department of Pharmacy, Shanghai Ninth People’s Hospital, Shanghai Jiao Tong University School of Medicine, Shanghai 200011, China; yanggangws@163.com (G.Y.); wangjing93wj@126.com (J.W.); gongchunai@163.com (C.G.);

**Keywords:** ionizing radiation, zeolitic imidazolate framework-8, kaempferol, KAE@ZIF-8

## Abstract

**Background/Objectives:** Kaempferol (KAE) is used to treat gamma radiation-induced damage. However, poor water solubility of KAE restricts its application. Therefore, we developed a KAE-loaded zeolitic imidazolate framework-8 (KAE@ZIF-8) to improve the solubility and bioavailability of KAE, thereby enhancing the radioprotective effect against gamma radiation. **Methods:** The composite was characterized using scanning electron microscopy (SEM), nitrogen adsorption/desorption analysis, X-ray diffraction (XRD), differential scanning calorimetry (DSC), equilibrium solubility assessments, in vitro release studies, stability evaluations, and drug-loading capacity measurements. The cytotoxic effects of KAE@ZIF-8 on Caco-2 cells were assessed in vitro. Meanwhile, the bioavailability of the preparation was also investigated. Finally, the protective efficacy of KAE@ZIF-8 against total body irradiation was evaluated in C57BL/6 mice. **Results:** The results indicated that KAE@ZIF-8 was successfully constructed, exhibiting a uniform hexagonal crystal morphology, with KAE transitioning from a crystalline to an amorphous state. As a carrier, ZIF-8 significantly enhanced the solubility of KAE by 9.2-fold, and the cumulative release within 12 h reached approximately 89%. Meanwhile, ZIF-8 could significantly enhance the bioavailability of KAE and reduce its toxicity. We found that pretreatment with KAE@ZIF-8 prolonged mouse survival time after 9 Gy total body irradiation (TBI). Mice were scarified on the 7th day after 7 Gy TBI. Results showed that KAE@ZIF-8 exhibited an improvement of the radioprotective effects, including weight loss mitigation, spleen index increase, radiation-induced intestinal injury attenuation, and modulation expression of IL-1β, IL-6, TNF-α and TGF-β1 following radiation. **Conclusions:** These results suggest the potential effect of ZIF-8 as an oral drug delivery carrier for radioprotective drugs.

## 1. Introduction

Ionizing radiation (IR) is a multifaceted tool in the field of oncology that serves as an indispensable component of cancer therapy. However, this carries a significant risk of collateral damage to healthy tissues [1,2]. The detrimental effects of IR, such as DNA strand breaks [3,4], oxidative stress [5], and systemic inflammation [6,7], are primarily mediated by reactive oxygen species (ROS) generated by water radiolysis, leading to acute radiation syndrome (ARS) and multi-organ dysfunction [8,9]. Despite advancements in shielding materials (e.g., lead aprons) and radioprotectors (e.g., amifostine) [10,11], challenges persist in achieving a balance among efficacy, biocompatibility, and practicality. Traditional shields are often heavy and inflexible, whereas synthetic radioprotectors are associated with toxicity concerns and have narrow therapeutic windows.

Natural flavonoids, such as kaempferol (KAE), have been shown to possess potent anti-oxidative and anti-inflammatory properties, which have the capacity to mitigate IR-induced DNA damage and apoptosis via Nrf2/ARE and NF-κB pathways [12,13]. However, the clinical translation of KAE is impeded by its poor bioavailability and rapid degradation [14,15]. Recently, zeolitic imidazolate framework-8 (ZIF-8) has emerged as a promising candidate for drug delivery because of its large specific surface area, high porosity, good pH sensitivity, good thermal stability, low toxicity and good biocompatibility [16]. It has been widely utilized as an effective carrier in the delivery of anti-tumor, antibacterial, and hypoglycemic agents [17,18,19]. Moreover, several previously encapsulated drugs with inherently poor water solubility, such as metronidazole, 5-fluorouracil, baicalin, and celastrol, have demonstrated significantly enhanced water solubility and bioavailability when loaded into ZIF-8 [20,21,22,23].

**Figure 1:**
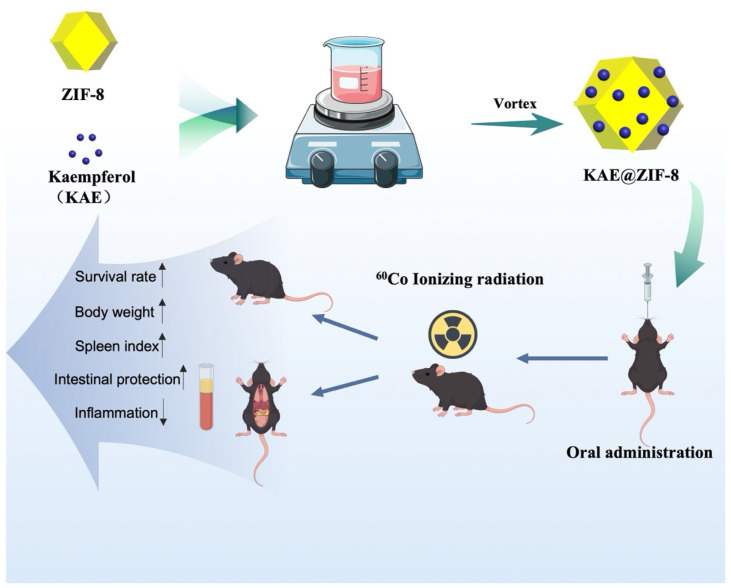
Schematic illustration of the formation of KAE@ZIF-8 and its radioprotective efficacy.

In this study, we propose a synergistic strategy for integrating ZIF-8′s structural advantages with the pharmacological benefits of KAE, evaluate the properties of zeolitic imidazolate frameworks (ZIF-8) nanoparticles for KAE, and investigate their synergistic radioprotective efficacy in vivo (Figure 1). By integrating materials science and pharmacology, we aspire to develop a next-generation platform for radiation protectants that is lightweight and multifunctional with applications in medicine, aerospace, and emergency scenarios.

## 2. Materials and Methods

### 2.1. Chemicals and Reagents

Zinc nitrate hexahydrate was bought from Shanghai McLin Bio-chemical Technology Co., Ltd. (Shanghai, China). 2-methylimidazole was purchased from Rhawn Chemical Technology Co., Ltd. (Shanghai, China). Methanol was obtained from Merck KGaA (Darmstadt, Germany), and kaempferol (purity ≥ 98%) was purchased from Dalian Meilun Biology Technology Co. (Dalian, China). Cell counting kit-8 was obtained from Beyotime Biotechnology (Shanghai, China). Sodium dodecyl sulfate, KH_2_PO_4_ and Na_2_HPO_4_ were obtained from Sinopharm Chemical Reagent (Shanghai, China)

### 2.2. Cell and Animals

Male C57BL/6 mice (6–8 weeks old, 20 ± 2 g) and male Sprague (SD) rats (6–8 weeks old, 200 ± 20 g) were purchased from Changzhou Cavens Experimental Animal Co. (Jiangsu, China). Human colorectal adenocarcinoma (Caco-2) cells were provided by the Cell Bank of Typical Culture Preservation Committee of the Chinese Academy of Sciences (SCSP-5027, Shanghai, China). All cells were cultured at 37 °C and 5% CO_2_. All mice and rats were managed under specific pathogen-free (SPF) conditions and were used according to the Guidelines of the National Institutes of Health on the Care and Use of Laboratory Animals. The animal experiments and feeding were conducted under specific pathogen-free (SPF) conditions, adhering to protocols approved by the Laboratory Animal Ethics Committee in Ninth People’s Hospital Affiliated to Shanghai Jiao Tong University School of Medicine (Ethics No. SH9H-2024-A1378-1).

### 2.3. High-Performance Liquid Chromatography (HPLC) Analytical Methods

The detection of KAE was performed on a Waters e2695 HPLC system (Waters Technologies, Milford, MA, USA). The chromatographic analysis conditions are explained below. The column used was a Diamonsil^®^ Plus C18 column (250 mm × 4.6 mm, 5 μm). The mobile phase consisted of methanol and phosphoric acid solution (0.4%) in a ratio of 55:45. The detection wavelength was set at 203 nm, and the flow rate was maintained at 1.0 mL/min. The column temperature was kept at 25 °C, and the injection volume was 20 μL. The quantified samples were filtered using a 0.45 µm filter membrane prior to automatic injection into the HPLC system.

### 2.4. KAE@ ZIF-8

#### 2.4.1. Preparation of KAE@ ZIF-8

KAE@ZIF-8 was synthesized using the solvent-adsorption method. Precise amounts of zinc nitrate and 2-methylimidazole hexahydrate were weighed. The two compounds were mixed in methanol until fully dissolved and then placed in a magnetic stirrer and stirred at 1000 rpm for 1.5 h to ensure an adequate reaction. Subsequently, the mixture was centrifuged at 12,000 rpm for 10 min and the supernatant was discarded. The resulting solid was collected, washed thrice with methanol, and vacuum-dried to obtain ZIF-8. Subsequently, specific amounts of ZIF-8 and KAE were dissolved in methanol by ultrasonication. The two solutions were combined, placed in a magnetic stirrer, and stirred at 1000 rpm while being protected from light for 12 h at room temperature. The resulting pellet was collected by centrifugation at 12,000 rpm for 10 min, washed thrice with methanol and twice with deionized water before being vacuum-dried. This process yielded KAE@ZIF-8.

#### 2.4.2. Drug Loading Time

A 10 mL solution of KAE in absolute ethanol with a concentration of 20 mg/mL was prepared and transferred to a 20 mL corked brown glass bottle. Subsequently, 500 mg of ZIF-8 was added to the bottle, which was placed on a magnetic stirrer. The resulting pellet was collected by centrifugation at 12,000 rpm for 10 min at various time intervals: 1, 2, 4, 6, and 12 h, while maintaining a water bath temperature of 55 °C and stirring at 300 rpm. The collected pellets were dried in an oven at 50 °C. The KAE content in each sample was determined using HPLC and drug loading was calculated. The drug loading-time curve for ZIF-8 was subsequently plotted.

#### 2.4.3. Drug Loading Concentration

10 mL of KAE anhydrous ethanol solutions with concentrations of 2, 5, 10, 30, and 50 mg/mL were prepared and placed in 20 mL stoppered brown glass bottles. Subsequently, 500 mg ZIF was added to the solution. The mixture was heated in a water bath at 55 °C and stirred at 300 rpm for 2 h. Afterward, it was centrifuged at 12,000 rpm for 10 min, eluted with absolute ethanol thrice, and dried in a precipitation oven at 50 °C. HPLC was employed to determine the KAE content of each sample, and drug loading was calculated.

#### 2.4.4. Drug Loading

KAE@ZIF-8 was accurately weighed and dispersed in an appropriate amount of ether. They were then subjected to ultrasonic treatment in a water bath for 10 min. Subsequently, the volume was adjusted to 5 mL with ether and centrifuged at 10,000 rpm for 5 min. The supernatant was diluted with methanol and subsequently analyzed using HPLC. Then, the measured peak area was recorded. The drug-loading of KAE@ZIF-8 was calculated as follows:

(1) $$DrugLoading\%=\frac{W_{KAE}}{W_{KAE@ZIF-8}}\times 100$$

W_KAE_ and W_KAE@ZIF-8_ represent the weights of KAE and KAE@ZIF-8 in the sample, respectively.

### 2.5. Sample Characterization

The surface topographies of ZIF-8 and KAE@ZIF-8 were examined using an S-4800 scanning electron microscope (Hitachi, Japan). A Nano ZS90 Zetasizer (Malvern, UK) was used to assess the particle size of samples. The samples were diluted with saline in a certain multiple before measuring the particle size. The specific surface areas and pore volumes of the samples were measured using an ASAP 2460 gas sorption analyzer (Micromeritics, Norcross, GA, USA) and their crystallographic properties were analyzed using an Ultima IV X-ray diffractometer (Rigaku, Japan). A DSC 3 differential scanning calorimeter (Metler-Toledo, Switzerland) was employed to monitor the changes in the temperature difference between the sample and the reference over time or temperature.

### 2.6. Equilibrium Solubility and In Vitro Release

Excess amounts of KAE and KAE@ZIF-8 samples were placed in 20 mL cermicillin vials and combined with 10 mL of purified water. The vials were secured in a water bath shaker, with the water bath temperature set to 25 °C and a rotation speed of 100 rpm for 72 h. After shaking for this duration to achieve equilibrium, the samples were centrifuged and filtered using a 0.45 μm hydrophilic membrane. The filtrate was then diluted with the same medium and the KAE concentration was determined by HPLC to be factored in the equilibrium solubility calculation.

The in vitro release profile of KAE was determined using the dialysis method. To simulate in vivo drug release following oral administration, three release media with varying pH were used to replicate the drug release process under different physiological conditions in the human body. KAE (5 mg) and an equivalent amount of KAE@ZIF-8 were placed in cellulose membrane dialysis bags (molecular cutoff, 8–14 kDa), and 1 mL of the release medium was added for dispersion. Initially, the dialysis bag was submerged in 50 mL of a pH 1.2 hydrochloric acid solution (containing 0.5% sodium lauryl sulfate, *w*/*w*) for 2 h at 37 °C with constant agitation at 100 rpm. Subsequently, the dialysis bag was transferred to PBS (pH 7.4) for a release period of 4 h. Finally, the pH of the PBS was adjusted to 6.8 using a small amount of phosphoric acid solution. At predetermined time points, samples were collected and an equal volume of release medium at the same temperature was added to maintain the conditions of the system. The samples were analyzed by HPLC, and the drug release curves for each sample were plotted.

### 2.7. Stability

KAE@ZIF-8 was subjected to accelerated testing at a temperature of 40 ± 2 °C and a relative humidity of 75 ± 5% for a duration of six months. Samples were collected at 0, 1, 2, 3, and 6 months to compare changes in particle size and drug loading before and after storage.

### 2.8. In Vivo Pharmacokinetic

12 male SD rats weighing 200 ± 20 g were selected and randomly divided into two groups for pharmacokinetic study. The rats were fasted for 12 h before the experiment but had free access to water. KAE powder and KAE@ZIF-8 were orally administered at doses of 20 mg/kg, respectively. At the predetermined time point, 500 microliters of blood were collected from the orbital vein and placed in a 1.5 mL heparinized centrifuge tube. The blood was centrifuged at 3000 rpm for 10 min to separate the plasma. Plasma was extracted three times using ether. The collected supernatant was dried with nitrogen at room temperature, and the residue was re-dissolved with 100 µL of methanol. Centrifuge the sample at 12,000 rpm for 10 min and inject 20 μL for HPLC analysis.

### 2.9. Cytotoxicity

The logarithmic growth phase Caco-2 cells were centrifuged in a centrifuge tube at 1000 rpm for 3 min. The original medium was discarded and an appropriate amount of fresh medium was added and mixed thoroughly. After counting, the cells were diluted to a concentration of 4 × 10^5^ cells/mL and seeded into a 96-well plate at a volume of 80 μL/well. Blank ZIF-8, KAE, and KAE@ZIF-8 were accurately weighed and diluted to various concentrations in culture medium. Three concentration gradients were established for each preparation, and 20 μL of each concentration was added to the corresponding wells. The 96-well plates were incubated for 24 h. Subsequently, 10 μL of CCK-8 solution (protected from light) was added to each well, and the plate was incubated for an additional 4 h. Absorbance was measured at 450 nm using a microplate reader to calculate the growth inhibition rate of Caco-2 cells.

### 2.10. Protective Effects of Ionizing Radiation

#### 2.10.1. Gamma Radiation and KAE@ZIF-8 Treatment

To investigate the radiation-protective effect of KAE@ZIF-8 on C57 BL/6 mice, the mice were randomly divided into eight groups. The pre-treatment groups were gavaged for one week, whereas the control and model groups received the same volume of normal saline. The groups were as follows.

(A) **Control group:** 9 mice and 5 mice, respectively, intragastrically administered saline, continuously dosed for 7 d before radiation with 0 Gy TBI.
(B) **9 Gy radiation group:** 9 mice, intragastrically administered saline, continuous dosing for 7 d before radiation with 9 Gy TBI.
(C) **9 Gy KAE 10 mg/kg group:** 9 mice, 10 mg/kg/day intragastrically administered KAE diluted with saline, continuous KAE dosing for 7 d before radiation with 9 Gy TBI.
(D) **9 Gy Blank ZIF-8 group:** 9 mice, 36.4 mg/kg/day intragastrically administered ZIF-8 diluted with saline, continuous ZIF-8 dosing for 7 d before radiation with 9 Gy TBI.
(E) **9 Gy KAE@ZIF-8 group:** 9 mice, 36.4 mg/kg/day intragastrically administered KAE@ZIF-8 diluted with saline, continuous KAE@ZIF-8 dosing for 7 d before radiation with 9 Gy TBI.
(F) **7 Gy radiation group:** 5 mice, intragastrically administered saline, continuous dosing for 7 d before radiation with 7 Gy TBI.
(G) **7 Gy KAE 10 mg/kg group:** 5 mice, 10 mg/kg/day intragastrically administered KAE diluted with saline, continuous KAE dosing for 7 d before radiation with 7 Gy TBI.
(H) **7 Gy KAE@ZIF-8 group:** 5 mice, 36.4 mg/kg/day intragastrically administered KAE@ZIF-8 diluted with saline, continuous KAE@ZIF-8 dosing for 7 d before radiation with 7 Gy TBI.

Mice were placed in holders and received a total dose of 7 or 9 Gy (1 Gy/min) whole-body ^60^Co gamma radiation at the Irradiation Center (Faculty of Naval Medicine, Second Military Medical University, Shanghai, China). The mice were monitored after irradiation. The 30-day survival rate in Groups A, B, C, D, and E was also determined. On the seventh day after 7 Gy irradiation, the mice in Groups A (5 mice), F, G, and H were anesthetized and euthanized. Body weight and spleen were recorded; meanwhile, serum was collected and frozen at −80 degrees Celsius until further testing. Additionally, intestinal tissues were embedded for pathological section detection.

#### 2.10.2. Organ Index

Mice were isolated on the 7th day after exposure to 7 Gy TBI, and the organ index was calculated using the following equation: organ index = (organ weight/body weight) × 100.

#### 2.10.3. Enzyme-Linked Immunosorbent Assay (ELISA)

The whole blood was centrifuged at 1000× *g* for 30 min. Following centrifugation, the sample of the serum supernatant was collected. The expression levels of IL-1β, IL-6, TNF-α, and TGF-β1 in the serum were measured using the IL-1β, IL-6, TNF-α, and TGF-β1 ELISA kit (Beyotime, China).

#### 2.10.4. Morphological Examination, Terminal Deoxynucleotidyl Transferase-Mediated d-UTP Nick-End Labeling (TUNEL) and Alpha-Smooth Muscle Actin (α-SMA) Assay

For histological analysis, intestines were meticulously dissected from mice in various treatment groups and fixed in 4% paraformaldehyde solution. The tissues were then embedded in paraffin, cut into 5 μm sections, and stained with hematoxylin and eosin (H&E). The sections were examined under a light microscope (Leica, Wetzlar, Germany) and the operator was blinded to the categorization of the subjects during the analysis. Villi and the number of crypts were observed within each circumference. Subsequently, apoptotic intestinal cells were identified by TUNEL staining (In Situ Cell Death Detection Kit, Roche, Basel, Switzerland). To evaluate the function of intestinal tissue, the expression of α-SMA on the intestine was detected by immunofluorescence. Then, the slides were immersed in a 200 mL working solution composed of sodium citrate antigen retrieval solution and deionized water in a microwave oven for antigen restoration, followed by incubation with α-SMA primary antibody (AF5466, Affinity biosciences, Jiangsu, China; 1:200) in a wet box overnight. A fluorescence microscope (AXIO SCOPE. A1, Carl Zeiss AG, Oberkochen, Germany) was used to distinguish the green fluorescence coming from the broken DNA and the red fluorescence coming from the α-SMA.

### 2.11. Statistical Analysis

All the results are expressed as the mean ± SD, and analysis was performed using SPSS 19.0 software. A significant discrepancy was identified among the various treatment groups, with more than three groups subjected to analysis. This discrepancy was investigated using one-way analysis of variance. Post hoc comparisons were performed using the Newman–Keuls Method. The Log-rank test was used to compare the survival curves. Statistical significance was set at *p* < 0.05.

## 3. Results

### 3.1. Preparation of Standard Curves

Different concentrations of KAE were prepared as follows: 0.5, 2, 5, 10, 30, 60, 90, and 150 µg/mL. The standard curve was represented by the equation *y* = 4911.9 *x* − 242.78, with a correlation coefficient of *r* = 0.999. These methods can be used to detect KAE.

### 3.2. Preparation of KAE@ZIF-8

**Figure 2:**
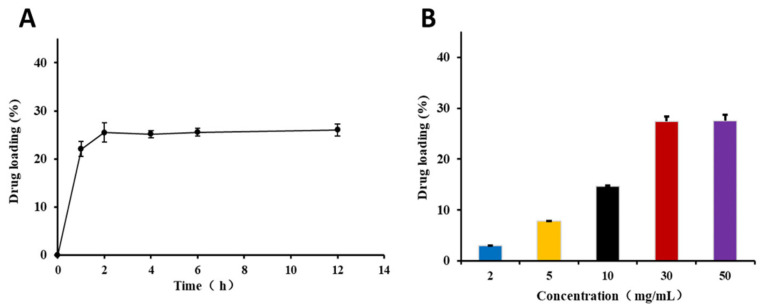
Optimization of the preparation process. (**A**) Influence of loading time on the drug loading of KAE@ZIF-8. Data are expressed as the mean ± SD. *n* = 3; (**B**) Impact of initial drug concentration on the drug loading of KAE@ZIF-8. Data are expressed as the mean ± SD. *n* = 3.

KAE@ZIF-8 was successfully synthesized via solvent adsorption. Under the conditions where the concentration of the KAE ethanol solution was 20 mg/mL and the drug loading temperature was set at 55 °C, the effect of drug loading time on the drug loading capacity is illustrated in Figure 2A. Drug loading reached a relatively high point at 2 h, and no significant differences were observed beyond this time point. Therefore, a drug-loading duration of 2 h is recommended.

Under ethanol solution incubation, an increase in the drug concentration significantly enhanced the drug-loading capacity of ZIF-8. Drug loading reached its maximum value at a concentration of 30 mg/mL (Figure 2B). Beyond this concentration, further increases in drug concentration did not result in additional drug loading, indicating that the system had reached saturation. Therefore, we recommended a drug loading concentration of 30 mg/mL. The final drug loading concentration was maintained at 30 mg/mL for a duration of 2 h, resulting in a drug loading of approximately 27.46 ± 0.85%.

### 3.3. Characterization of KAE@ZIF-8

**Figure 3:**
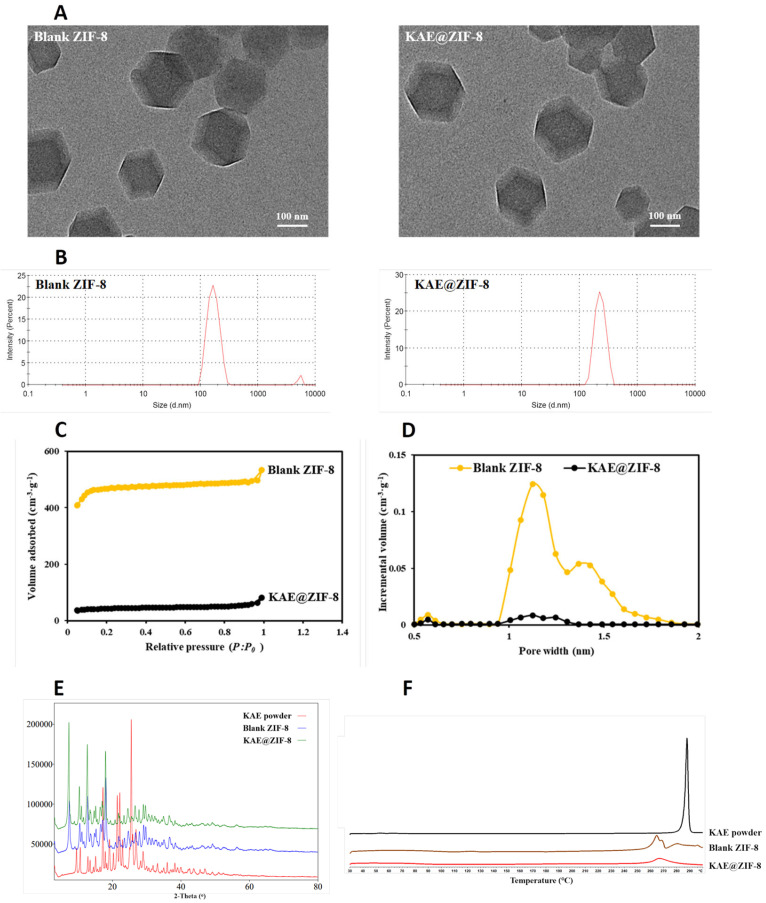
Characterization of samples. (**A**) Scanning electron microscopy (SEM) image of blank ZIF-8 and KAE@ZIF-8; (**B**) Particle size of blank ZIF-8 and KAE@ZIF-8; (**C**) Nitrogen adsorption isotherms comparing blank ZIF-8 and KAE@ZIF-8; (**D**) Pore size distribution profiles for blank ZIF-8 and KAE@ZIF-8; (**E**) X-ray diffraction (XRD) patterns of blank ZIF-8, KAE powder, and KAE@ZIF-8; (**F**) Differential scanning calorimetry (DSC) thermograms of blank ZIF-8, KAE powder, and KAE@ZIF-8.

Scanning electron microscopy (SEM) images of blank ZIF-8 and KAE@ZIF-8 are presented in Figure 3A, revealing that both samples consisted of homogeneous cubic crystals with a particle size of approximately 170 nm. The particle size and morphology of ZIF-8 remained consistent with those of the blank ZIF-8 after loading with KAE, indicating that the drug-loading process did not affect the particle size or morphology of ZIF-8. The particle size (Figure 3B) measurement revealed that the particle size of blank ZIF-8 is similar to that of KAE@ZIF-8, which is consistent with the SEM result. The particle sizes of blank ZIF-8 and KAE@ZIF-8 were 183.1 nm (PDI = 0.293) and 191.3 nm (PDI = 0.268), respectively. The particle size measured by the particle size analyzer were slightly larger than that observed in the SEM, which might be due to the agglomeration effect of nanoparticles in saline.

The specific surface areas and pore volumes of the samples were measured using a nitrogen adsorption/desorption instrument. The BET specific surface area of blank ZIF-8 was 1527.7 m^2^/g, and the volume of desorption accumulated pores was 0.583 cm^3^/g. Figure 3C illustrates that blank ZIF-8 exhibits sharp N_2_ adsorption in the low-pressure region, confirming its micropore adsorption characteristics. However, the specific surface area of KAE@ZIF-8 decreased significantly after KAE loading, indicating that the pores and surface of ZIF were occupied and strongly adsorbed by drug molecules. Figure 3D illustrates a marked reduction in the pore volume of ZIF after drug loading, which aligns with the findings reported in the literature on ZIF drug loading [21].

Crystallographic analysis of the samples using X-ray diffraction (XRD) demonstrated that the XRD patterns indicated changes in the crystal properties of KAE before and after loading with ZIF-8 (Figure 3E). The KAE powder was found to exist in a stable crystalline form, exhibiting significant diffraction peaks at 17.22, 21.42, 22.14, and 25.48 °. However, the characteristic diffraction peaks of KAE completely disappeared for the KAE@ZIF-8 composite after drug loading. This observation suggests that KAE transitioned to an amorphous form within ZIF-8, indicating that KAE was encapsulated by ZIF-8 at the molecular level, resulting in a change from a crystalline to an amorphous state [24]. The characteristic diffraction peaks of blank ZIF-8 remained evident after drug loading, but the intensity of this peak decreased, likely because of the high drug loading.

To further verify the physical incorporation of KAE into ZIF-8, the thermal behavior of KAE following its loading into ZIF-8 was analyzed using DSC. As illustrated in Figure 3F, KAE exhibits a melting point endothermic peak at 287.833 °C, indicating that the KAE feedstock exists in crystalline form, which is consistent with previously reported values in the literature [25]. Concurrently, ZIF-8 displayed a melting point endothermic peak at 266.667 °C. In the DSC spectrum of KAE@ZIF-8, the endothermic peak corresponding to KAE disappeared, leaving only the endothermic peak of blank ZIF-8. This observation suggests that the structural orientation of the KAE crystal was altered by the successful encapsulation of KAE within the ZIF-8 pores. It is evident that the KAE molecules are distributed in an amorphous state within ZIF-8, which is corroborated by the XRD results. Furthermore, the presence of KAE in the amorphous form of KAE@ZIF-8 was anticipated to enhance drug release and improve in vivo absorption [26].

### 3.4. Solubility, Stability and Cytotoxicity of KAE@ZIF-8

**Figure 4:**
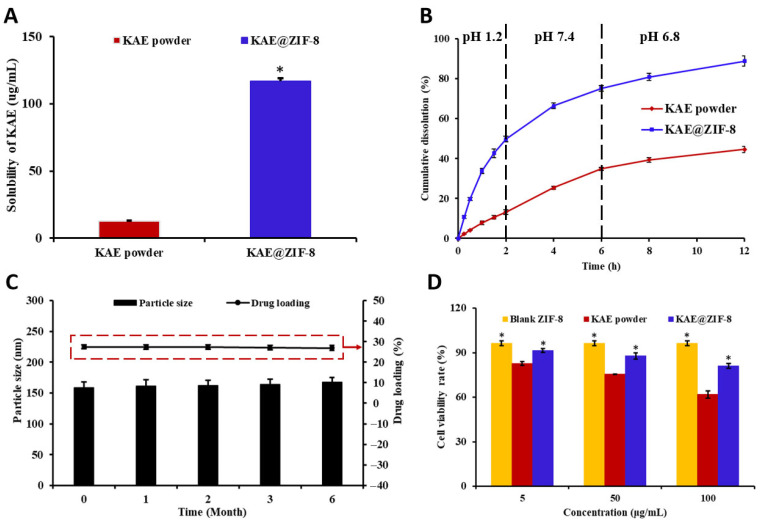
Studies on solubility, stability, and cytotoxicity of samples. (**A**) Apparent solubility measurements of KAE powder and KAE@ZIF-8 (*p < 0.05 vs. KAE powder). Data are expressed as the mean ± SD. *n* = 3; (**B**) Cumulative drug release profiles comparing KAE powder and KAE@ZIF-8. Data are expressed as the mean ± SD. *n* = 3; (**C**) Stability evaluation of particle size and drug loading efficiency (Rectangular region delineated by a red dotted line) of KAE@ZIF-8 over a 6-month period under accelerated conditions (40 ± 2 °C, 75 ± 5% relative humidity). Data are expressed as the mean ± SD. *n* = 3; (**D**) Cytotoxicity analysis of blank ZIF-8, KAE powder, and KAE@ZIF-8 on Caco-2 cells (*p < 0.05 vs. KAE powder). Data are expressed as the mean ± SD. *n* = 3.

The solubility of a drug in physiological media is a critical factor in determining its bioavailability [27]. KAE is classified as a poorly water-soluble drug because of the influence of lattice energy. By utilizing the steric hindrance effect of the ZIF pores, the spatial orientation of the KAE molecules and the intermolecular forces necessary for recrystallization were effectively restricted. This limitation hinders the transformation of amorphous KAE into crystalline forms, while simultaneously enhancing the loading capacity and efficiency of the drug, thereby increasing the solubility of KAE. The equilibrium solubility results are illustrated in Figure 4A. The apparent solubility of KAE@ZIF-8 was 9.2-fold greater than that of KAE powder. The mechanism by which ZIF-8-loaded KAE enhances the equilibrium solubility of the drug may be attributed to a bimolecular process in which KAEs form nanoclusters within the large cavity of ZIF-8. This phenomenon has been theoretically and experimentally validated in several previous studies on ZIF-8 delivery [28].

In addition to solubility, the investigation of dissolution and dissolution rates is of great significance for evaluating pharmaceutical preparations. To simulate drug release from each group of preparations in the human digestive tract, in vitro release studies were conducted using release media with varying pH levels. During the first 2 h, a pH 1.2 hydrochloric acid solution was employed to replicate the environment of the human gastric juice. For the subsequent 4 h, a medium with pH 7.4 was used to mimic the pH conditions of human intestinal fluid (ranging from pH 6.0 to 8.0). Finally, a medium with pH 6.8 was used to simulate the neutral pH environment of the large intestine in the lower part of the digestive tract. The in vitro release curves of KAE powder and KAE@ZIF-8 are shown in Figure 4B. In the simulated gastric juice release medium with pH 1.2, KAE powder demonstrated a lower cumulative drug release of approximately 13% over the first 2 h. In contrast, KAE@ZIF-8 exhibited relatively rapid drug release during the same period, with a cumulative release of approximately 49% within the first 2 h. After 4 h of continuous release in a medium with pH 7.4, the cumulative release of KAE powder and KAE@ZIF-8 was approximately 35% and 75%, respectively. In the medium with pH 6.8, the cumulative release of KAE increased gradually over time, with the cumulative release of KAE@ZIF-8 reaching approximately 89% after 12 h, whereas the cumulative release of KAE was 45%. Therefore, ZIF-8 significantly enhanced cumulative dissolution and drug release rates following drug loading.

Stability is a crucial parameter in the assessment of pharmaceutical preparations. This study evaluated the stability of KAE@ZIF-8 by analyzing the changes in particle size and drug loading. As illustrated in Figure 4C, after six months of storage, both the drug loading and particle size of KAE@ZIF-8 showed minimal changes compared with the initial measurements at zero months. The particle size and drug loading remained largely unchanged, indicating that KAE@ZIF-8 exhibited good stability. Furthermore, the KAE@ZIF-8 suspension in saline exhibited no significant agglomeration or precipitation within several hours after preparation, suggesting its ability to maintain a relatively stable dispersion over a short period. Moreover, after storage for 6 months, the KAE@ZIF-8 powder was successfully re-dispersed in saline solution and retained good dispersibility and suspension stability, indicating that the powder formulation also possesses favorable long-term stability within the 6-month timeframe.

KAE powder induces significant gastrointestinal irritation. Therefore, we selected Caco-2 cells, which closely resemble the structure and function of differentiated small intestinal epithelial cells, to investigate the cytotoxic effects of the various preparations on these cells. The results are presented in Figure 4D, demonstrating that the survival rate of Caco-2 cells exceeded 95% after 24 h of co-incubation with blank ZIF-8, indicating no significant cytotoxic effects. The cytotoxicity of KAE powder on Caco-2 cells was positively correlated with the drug concentration, with a survival rate of only approximately 60% at a concentration of 100 µg/mL. However, after formulating KAE@ZIF-8, the cell viability remained above 80% at the same drug concentration of 100 µg/mL, suggesting that ZIF-8 loading effectively mitigates the cytotoxicity of KAE powder.

### 3.5. Pharmacokinetics Study

**Figure 5:**
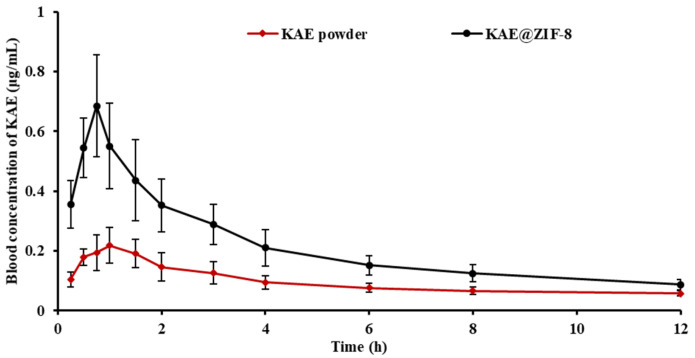
Mean plasma concentration-time profiles of KAE powder and KAE@ZIF-8 in SD rats after oral administration. Data are expressed as the mean ± SD. *n* = 6.

To further investigate the effect of ZIF-8 on the bioavailability in vivo of KAE, we evaluated the pharmacokinetic behavior of KAE powder and KAE@ZIF-8 in male SD rats. The mean plasma concentration-time profile is presented in Figure 5, and the corresponding pharmacokinetic parameters are summarized in Table 1. The peak plasma concentration (C_max_) and area under the plasma concentration-time curve (AUC_0-∞_) in the KAE@ZIF-8 group were significantly higher than those in the KAE powder group. Specifically, the C_max_ and AUC_0–∞_ values of the KAE@ZIF-8 were 3.05 times and 2.23 times higher, respectively, than those of the KAE powder group. These findings indicate that KAE@ZIF-8 can substantially enhance the oral bioavailability of KAE. Additionally, the time to reach the peak plasma concentration (T_max_) of the KAE@ZIF-8 group was observed at 0.96 h, which was earlier than that in the KAE powder group. This observation may be attributed to the rapid release of KAE from KAE@ZIF-8 in gastric juice, a phenomenon that aligns with the result of the in vitro release study.

### 3.6. KAE@ZIF-8 Protects Mice Against Radiation-Induced Motility and Weight Reduction

**Figure 6:**
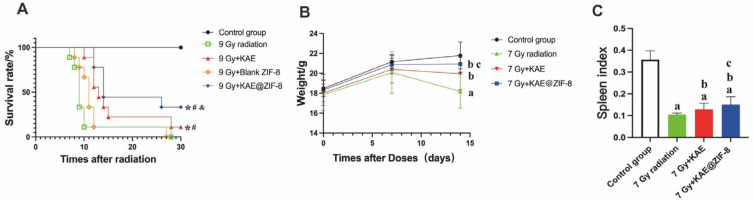
(**A**) KAE@ZIF-8 protects mice against radiation-induced death. Data are expressed as the mean ± SD. *n* = 9; * p < 0.05 (vs. Control group), ^#^ *p* < 0.05 (vs. 9 Gy radiation) ^&^ *p* < 0.05 (vs. 9 Gy + KAE group). (**B**) KAE@ZIF-8 inhibits mouse weight loss. Data are expressed as the mean ± SD. *n* = 5, ^a^ *p* < 0.05 (7 Gy radiation vs. Control group), ^b^ *p* < 0.05 (7 Gy + KAE vs. 7 Gy radiation; 7 Gy + KAE@ZIF-8 vs. 7 Gy radiation), ^c^ *p* < 0.05 (7 Gy + KAE@ZIF-8 vs. 7 Gy + KAE). (**C**) KAE@ZIF-8 increases organ index. Data are expressed as the mean ± SD. *n* = 5, ^a^ *p* < 0.05 (7 Gy radiation vs. Control group), ^b^ *p* < 0.05 (7 Gy + KAE vs. 7 Gy radiation; 7 Gy + KAE@ZIF-8 vs. 7 Gy radiation), ^c^ *p* < 0.05 (7 Gy + KAE@ZIF-8 vs. 7 Gy + KAE).

To evaluate the radiation protection effect of KAE@ZIF-8, we first plotted the survival rate over a 30-d period following exposure to a lethal dose of 9 Gy TBI (Figure 6A). The survival rate of the control group was 100%, whereas the groups with radiation injury decreased sharply over time, indicating significant radiation damage. However, following drug intervention (with KAE, blank ZIF-8, and KAE@ZIF-8), the survival rate of the blank ZIF-8 group was 0%, showing no significant difference compared to 9 Gy radiation group, suggesting that the blank vector did not provide protective effect. The median survival duration in the KAE 10 mg/kg group was 13 d, which was lower than that in the KAE@ZIF-8 dosing group (14 d). This indicates that KAE has radiation protective properties. The survival rate of the KAE@ZIF-8 group (33.3%) was significantly higher than that in the other intervention groups and 9 Gy radiation group (*p* < 0.05). As shown in Figure 6B, compared to weight baseline (D7), weight loss occurred in all groups after receiving 7 Gy total body ionizing radiation compared to the control group (3.35 ± 3.23)%. In comparison to the 7 Gy radiation group (−9.29 ± 1.89)%, both KAE and KAE@ZIF-8 pretreatment are all significantly reduced body weight loss (−2.24 ± 1.45)% and (0.16 ± 0.58) %, respectively (*p* < 0.05, *p* < 0.05). These results indicate that KAE has protective effects against ionizing radiation and that the nano-drug delivery system enhances the radiation protective efficacy of KAE.

### 3.7. KAE@ZIF-8 Reduces Ionizing Radiation-Induced Spleen Injury in Mice

To investigate the effect of KAE@ZIF-8 on the immune organs of mice following ionizing radiation, we sacrificed the mice that received 7 Gy TBI on the seventh day post-exposure. We then removed the spleens, weighed them, and calculated the spleen index. As shown in Figure 6C, the spleen index of the 7 Gy radiation group was significantly lower than control group (*p* < 0.05), indicating that radiation caused atrophy of the immune organs. Compared to the 7 Gy radiation group, both KAE and KAE@ZIF-8 had a significantly increased spleen index (*p* < 0.05, *p* < 0.05). Notably, the spleen index of the 7 Gy KAE@ZIF-8 group was higher than 7 Gy KAE group (*p* < 0.05). These results suggest that KAE effectively protected the function of immune organ and alleviated radiation-induced splenic atrophy, whereas KAE@ZIF-8 enhanced the radioprotective effects of KAE on immune organ.

### 3.8. KAE@ZIF-8 Attenuates Ionizing Radiation-Induced Small Intestinal Tissue Damage in Mice

**Figure 7:**
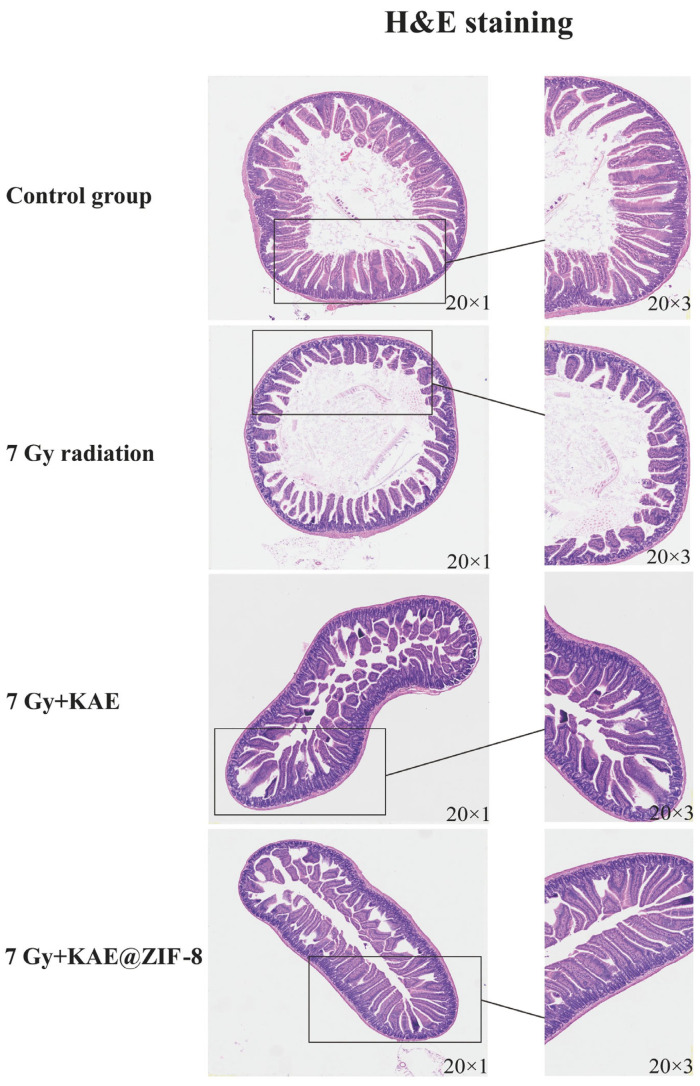
Representative micrographs of H&E-stained sections of the intestinal tissue (20 × 1; 20 × 3). KAE@ZIF-8 pretreatment significantly increases the protective efficacy of KAE against radiation-induced damage in intestinal tissues.

To evaluate the effect of KAE@ZIF-8 on the intestinal tissue of mice following ionizing radiation, the mice were sacrificed on the 7th d after receiving 7 Gy TBI. H&E staining (Figure 7) indicated that the intestinal structure of the control group with normal morphology, including the mucosal layer, villi, and crypts. In contrast, significant pathological damage, including villus atrophy, destruction of crypt structures, and necrosis of the mucosal layer, was observed in the intestines of the radiation group after 7 d exposure to 7 Gy. Intestinal damage was partially alleviated in the group receiving 10 mg/kg KAE, with a reduction in inflammation compared to the radiation group. However, local destruction of crypt structures and villus shortening persisted. In the group that was administered KAE@ZIF-8, the intestinal structure showed significant improvement, with the villi and crypts remaining largely intact in morphology. These results suggest that the KAE@ZIF-8 nanocarriers can significantly enhance the protective effect of KAE against radiation-induced intestinal tissue injury.

**Figure 8:**
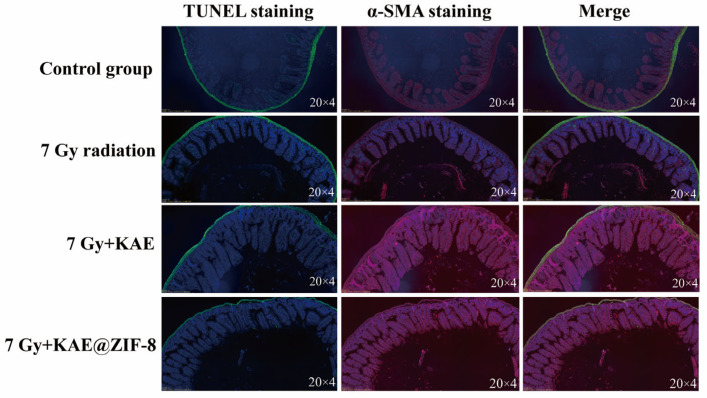
Representative micrographs of TUNEL/α-SMA staining and merged sections of the intestinal tissue (20 × 4). KAE@ZIF-8 inhibits radiation-induced intestinal apoptosis and intestinal dysfunction in mice.

To further evaluate the effects of KAE@ZIF-8 on apoptosis and the structure of intestinal tissue after ionizing radiation, TUNEL (green fluorescence) and α-SMA (red fluorescence) double staining were performed on small intestinal tissue. As shown in Figure 8, compared to the control group, the number of apoptotic cells in the villi and crypts was significantly increased in 7 Gy radiation group; meanwhile, the α-SMA immunofluorescence signal intensity in 7 Gy radiation group was notably disrupted. After the administration of KAE and KAE@ZIF-8, the number of apoptotic cells was significantly reduced, and α-SMA levels were markedly restored. These results suggest that KAE has a protective effect on the intestinal tissues of mice following ionizing radiation and KAE@ZIF-8 can effectively enhance the inhibitory effect of KAE on radiation-induced intestinal cell apoptosis and dysfunction.

### 3.9. KAE@ZIF-8 Attenuates Ionizing Radiation-Induced Inflammation in Mice

**Figure 9:**
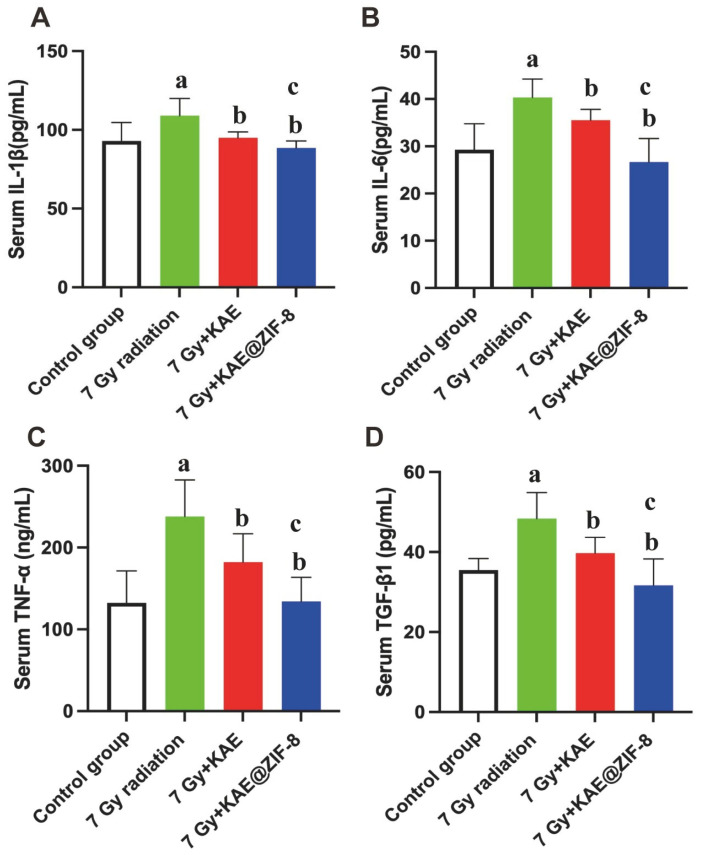
KAE@ZIF-8 suppresses the abnormal expression levels of IL-1β (**A**), IL-6 (**B**), TGF-β1 (**C**), and TGF-α (**D**) in serum after 7 Gy radiation. Data are expressed as the mean ± SD. *n* = 5, ^a^ *p* < 0.05 (7 Gy radiation vs. Control group), ^b^ *p* < 0.05 (7 Gy + KAE vs. 7 Gy radiation; 7 Gy + KAE@ZIF-8 vs. 7 Gy radiation), ^c^ *p* < 0.05 (7 Gy + KAE@ZIF-8 vs. 7 Gy + KAE).

To investigate the effect of KAE@ZIF-8 on the inflammatory response induced by ionizing radiation, mice were sacrificed on the seventh day after exposure to 7 Gy whole-body ionizing radiation. Serum was collected, and the serum levels of inflammatory factors were measured. As shown in Figure 9A-D, ionizing radiation significantly increased the levels of IL-1β, IL-6, TNF-α, and TGF-β1 compared to the control group (*p* < 0.05, *p* < 0.05, *p* < 0.05, *p* < 0.05). In comparison to the 7 Gy radiation group, the levels of IL-1β, IL-6, TNF-α, and TGF-β1 were reduced in both the 7 Gy KAE and 7 Gy KAE@ZIF-8 group (*p* < 0.05, *p* < 0.05, *p* < 0.05, *p* < 0.05). Furthermore, the 7 Gy KAE@ZIF-8 group exhibited a significant reduction in the levels of IL-1β, IL-6, TNF-α, and TGF-β1 compared to the 7 Gy KAE group (*p* < 0.05, *p* < 0.05, *p* < 0.05, *p* < 0.05). These results suggest that KAE can alleviate the inflammatory response caused by ionizing radiation and KAE@ZIF-8 can effectively enhance the efficacy of KAE in reducing inflammatory response.

## 4. Discussion

Ionizing radiation is frequently encountered during nuclear accidents, medical radiodiagnoses, and therapeutic interventions [29]. Radiotherapy is a critical modality for the treatment of malignant tumors in humans [30]. Despite its efficacy in targeting malignant neoplasms, this therapeutic approach damages normal tissues [31]. Acute radiation syndrome commonly manifests in the intestinal [32], hematopoietic [33], and immune system [5] following exposure. These toxic effects are predominantly mediated by mechanisms involving apoptosis, oxidative stress, and inflammation. Flavonoids have a potent antioxidant effect due to their high redox potential, which allows them to act as hydrogen donors and reducing agents, metal chelating potential as well as quenchers of singlet oxygen. Including Quercetin, Hesperidin, Rutin et al., which have been claimed with radioprotective properties [34]. KAE possesses antioxidant, anti-inflammatory, and immunomodulatory activities in both in vitro and vivo radiation injury models. Empirical evidence has demonstrated that KAE mitigates radiation-induced tissue damage and enhances cell survival. Its protective actions include the scavenging of intracellular free radicals, restoration of circulating endothelial progenitor cell populations, and normalization of aberrant expression levels of Prx5 and caspase 3 and caspase 9 at both the mRNA and protein levels, thereby attenuating radiation-induced tissue injury and apoptosis [35]. Furthermore, research suggests that KAE confers neuroprotection against cerebral ischemia/reperfusion (I/R) injury in vivo. The underlying mechanism is primarily attributed to its capacity to shield the brain from I/R-induced oxidative stress, inflammation, and apoptosis through the upregulation of phosphorylated Akt (p-Akt) and nuclear factor erythroid 2-related factor 2 (Nrf-2), alongside the downregulation of phosphorylated nuclear factor kappa B (p-NF-κB) and glycogen synthase kinase-3 beta (p-GSK-3β) [36].

The utilization of KAE is limited by its poor bioavailability and rapid degradation [37]. Metal–organic frameworks (MOFs) are 3-dimensional porous structures with a cage-like conformation. The zeolitic imidazolate framework-8 (ZIF-8) MOFs, which are built by coordination between Zn^2+^ and dimethylimidazole, exhibit excellent biocompatibility and can enable the controlled delivery and release of cargos of interest while improving the bioavailability of drugs. Zhang et al. reported that tannic acid and zinc ion coordination nanoparticles can promote intestinal mucosal healing for treatment of inflammatory bowel disease [38]. Although the pharmacological dosage of Zn^2+^ can be used to enhance intestinal barrier function [39]. However, high concentrations of zinc may have side effects on healthy tissues [40], which requires our special attention and emphasis. The metal–organic framework ZIF-8, characterized by its adjustable porosity, elevated specific surface area [41], and capacity to integrate high-atomic-number elements [42] (e.g., bismuth and tungsten) to improve X- and gamma-ray attenuation, has emerged as a promising delivery vehicle. He et al. found a ZIF-8-based sitagliptin-release platform for multi-effective radiation-induced intestinal injury targeting therapy and intestinal flora protective capabilities. Mechanistically, the radioprotective effects may be associated with drug loading and increased Zn^2+^ levels, a synergistic role of the ZIF-8 carrier. Increasing the Zn^2+^ level in the gastrointestinal tract can suppress inflammation, mitigate oxidative injury, and remediate gastrointestinal dysbiosis [43].

In this study, KAE was effectively encapsulated in ZIF-8 using a solvent incubation technique to form a nanoscale drug delivery system (KAE@ZIF-8). This system exhibited notable stability. In vitro analyses revealed that the encapsulated KAE predominantly existed in an amorphous state, displaying an apparent solubility that was approximately 9.2-fold greater than that of the unencapsulated KAE. The drug-loading process markedly enhanced the cumulative dissolution and release rates of KAE. An enhanced dissolution of KAE can significantly influence its bioavailability. Pharmacokinetic results further corroborated this finding, confirming that KAE@ZIF-8 effectively enhances the oral bioavailability of KAE. Notably, at a concentration of 100 μg/mL, KAE@ZIF-8 increased the viability of Caco-2 cells by over 20% relative to KAE powder, thereby effectively mitigating the cytotoxic effects of KAE powder on Caco-2 cells. In vivo studies revealed that KAE@ZIF-8 effectively protected the mice against the damage of a lethal dose of whole-body radiation and mitigated radiation-induced weight loss. Consistent with the typical manifestations of acute radiation syndrome, immune system impairment was observed, as evidenced by a marked reduction in the spleen index following radiation exposure. KAE treatment significantly increased the spleen index. Notably, the KAE@ZIF-8 treatment group exhibited more marked attenuation of radiation-induced spleen index decline than the KAE group, indicating its superior efficacy in preserving immune organ function and preventing radiation-induced splenic atrophy. Whole-body radiation doses ranging from 6 to 9 Gy are typically associated with intestinal radiation injury [44,45]. The gastrointestinal system exhibits high sensitivity to radiation. Radiation-induced gastrointestinal syndromes are the primary causes of mortality after radiation exposure. In this study, histopathological alterations, apoptosis, and intestinal functionality in the small intestines of irradiated mice were assessed using H&E staining, TUNEL staining, and α-SMA staining. Histological analysis revealed that radiation exposure severely compromised the structural integrity of the small intestine, as evidenced by the reduction in the villus height and decrease in crypt numbers. Administration of KAE ameliorated these effects by enhancing the integrity and length of the small intestinal villi and increasing the crypt counts. TUNEL staining demonstrated that radiation markedly elevated apoptosis in both villus and crypt cells. Notably, the KAE@ZIF-8 formulation exhibited superior efficacy compared to KAE powder in mitigating damage to the small intestinal villi and crypts, reducing radiation-induced apoptosis, and preserving intestinal function. Given that inflammatory responses are critical mediators of ionizing radiation-induced injury, serological analyses were conducted, revealing that KAE@ZIF-8 attenuated inflammation more effectively than KAE powder to reduce radiation-induced elevations of IL-1β, IL-6, TNF-α, and TGF-β1 levels. Experiments involving larger sample sizes and long-term toxicity and effect of ZIF-8 carrier will be designed in the future. Collectively, these findings suggested that KAE@ZIF-8 enhanced the radioprotective properties of KAE more effectively than the free form.

## 5. Conclusions

A solvent incubation technique was employed to synthesize stable KAE@ZIF-8, which significantly improved the solubility and bioavailability of KAE. In vivo assessments demonstrated that KAE@ZIF-8 exhibited markedly superior protective efficacy compared with KAE powder, as evidenced by increased survival rates following radiation exposure, mitigation of spleen atrophy and intestinal injury, and suppression of inflammatory cytokines. These findings advance KAE@ZIF-8 from a conceptual “potential technology” to a “mechanistically elucidated and translationally viable” approach for radiation protection.

## Figures and Tables

**Table 1:** Main pharmacokinetic parameters of the indicated preparations in SD rats after oral administration. Data are expressed as the mean ± SD. *n* = 6.

Formulation of KAE	T_max_ (h)	C_max_ (μg/mL)	AUC_0-∞_ (μg/mL·h)
KAE powder	1.65 ± 0.80	0.25 ± 0.05	1.14 ± 0.03
KAE@ZIF-8	0.96 ± 0.29	0.76 ± 0.10 **	2.54 ± 0.08 **

C*_max_*, peak plasma concentration; T*_max_*, time to reach the peak plasma concentration; AUC_0–∞_, area under the plasma concentration-time curve. ** p < 0.05 vs. KAE powder.

## Data Availability

The original contributions presented in this study are included in the article. Further inquiries can be directed to the corresponding author.
